# Triphenyl Phosphine-Functionalized Chitosan Nanoparticles Enhanced Antitumor Efficiency Through Targeted Delivery of Doxorubicin to Mitochondria

**DOI:** 10.1186/s11671-017-1931-1

**Published:** 2017-02-28

**Authors:** Jiahui Hou, Xiwei Yu, Yaping Shen, Yijie Shi, Chang Su, Liang Zhao

**Affiliations:** 1 School of Pharmacy, Jinzhou Medical University, Jinzhou, 121000 People’s Republic of China; 2 School of Veterinary Medicine, Jinzhou Medical University, Jinzhou, 121000 People’s Republic of China

**Keywords:** Doxorubicin, Nanoparticles, Chitosan, Mitochondria, Cytotoxicity

## Abstract

Mitochondria as an important organ in eukaryotic cells produced energy through oxidative phosphorylation and also played an important role in regulating the apoptotic signal transduction process. Importantly, mitochondria like nuclei also contained the functional DNA and were very sensitive to anticancer drugs which could effectively inhibit the synthesis of nucleic acid, especially the production of DNA. In this work, we designed novel triphenyl phosphine (TPP)-conjugated chitosan (CS) nanoparticles (NPs) for efficient drug delivery to cell mitochondria. The results showed that compared with free doxorubicin (Dox), Dox-loaded TPP-NPs were specifically distributed in mitochondria of tumor cells and interfered with the function of mitochondria, thus resulted in the higher cytotoxicity and induced the significant cell apoptosis effect. Taken together, triphenyl phosphine-conjugated chitosan nanoparticles may become a promising mitochondria-targeting nanocarrier candidate for enhancing antitumor effects.

## Background

Doxorubicin (Dox) was an anthracycline glycoside antibiotic drug derived by chemical semisynthesis from a bacterial species and widely clinically used in the hydrochloride form. It was commonly used in the treatment of a wide range of cancers, including hematological malignancies (blood cancers, like leukemia and lymphoma), many types of carcinoma (solid tumors), and soft tissue sarcomas [1–6]. Although Dox effectively killed tumor cells and showed the excellent clinical effect in the treatment of cancer, significant systemic adverse reactions were also emerged such as bone marrow suppression, congestive heart failure, and typhlitis [7–11]. Especially, after a time period of drug exposure, the treatment response to chemotherapy in some patients was highly declined due to an effect known as acquired resistance [12, 13]. Therefore, it was necessary to find an efficient treatment strategy on the application of Dox for enhancing cytotoxicity to tumor tissues and overcoming drug resistance. In recent years, liposomal Dox (liposome doxorubicin) and Dox-loaded nanoparticles enhanced the intracellular accumulation of Dox or changed the cellular distribution of drug, thus strengthening the cytotoxicity of anticancer drugs and limiting the cardiotoxicity of Dox [14–20]. Mitochondria as the main provision of cellular power played a vital role on cell survival and death, and it not only produced most ATP which the cell metabolism required but also regulated metabolism and programmed cell death. Some anticancer drugs directly interfered with mitochondrion respiratory chains and eventually led to tumor cell death [21]. Therefore, dysfunction of mitochondria would be a preferential target of many antineoplastic agents [22–25]. It was reported that the anticancer drug Dox-loaded nanocarrier delivered Dox to mitochondria in a targeted manner to interact with mtDNA, leading to the direct dysfunction of mitochondria in cancer cells. Accumulation of Dox in mitochondria generated excessive reactive oxygen species and caused damage to the mitochondrial respiratory chain, further resulting in mitochondrial lipid oxidation and increasing release of cytochrome c [26]. Chitosan (CS) was a natural cationic polymer with the excellent biocompatibility, low toxicity, and especially low cost compared to other nano carriers. CS had the positive charges and easily combined with the negatively charged membrane, facilitating its internalization. As membrane potential of mitochondria was much higher than other organelles because of ATP synthesis and ion transport, therefore, triphenyl phosphine (TPP) as a kind of lipophilic cationic compound could be effectively targeted in mitochondria. Combination of CS and TPP could smartly target the loaded drug to mitochondria, thus triggering programmed cell death process, leading to cell stress response, cytochrome c release, and then the damage and cytotoxicity of the tumor cells.

Herein, we designed novel triphenyl phosphine (TPP)-conjugated chitosan nanoparticles (TPP-CS NPs) for the targeted mitochondrial delivery of Dox to enhance antitumor efficiency. Triphenyl phosphine was grafted onto the surface of (as-prepared) chitosan nanoparticles for targeting the mitochondria, and Dox was encapsulated into NPs for investigating the drug loading and in vitro release process. Furthermore, the cellular distribution, in vitro cytotoxicity, and cellular apoptosis were also investigated to evaluate cancer treatment efficacy by the targeted mitochondrial delivery of Dox via triphenyl phosphine-conjugated chitosan nanoparticles.

## Methods

### Materials

Chitosan (CS) of medium molecular weight (deacetylation degree, 80%; molecular weight, 400,000) was purchased from Haixin Biological Product Co., Ltd (China); Dox hydrochloride was purchased from Beijing Huafeng Lianbo Technology Co., Ltd. (China). 3-(4,5-Dimethylthiazol-2-yl)-2,5-diphenyltetrazolium bromide (MTT), 1-ethyl-3-(3-dimethylaminopropyl) carbodiimide (EDAC), triphenyl phosphine (TPP), and *N*-hydroxysuccinimide (NHS) were obtained from Sigma-Aldrich Co. (St Louis, MO, USA); A549 cell lines and Hela cells were purchased from the Institute of Biochemistry and Cell Biology of Chinese Academy of Sciences (Shanghai, People’s Republic of China). All other chemicals purchased were of analytical grade and were obtained from a variety of vendors.

### Preparation and Characterization of Dox-Loaded TPP-CS NPs

According to the previous reported literature [27], Dox-loaded triphenyl phosphine-conjugated chitosan nanoparticles were prepared by ionic crosslinking method. Briefly, 10 mL stock solution of Dox at a concentration of 1 mg/mL was pre-mixed with CS solution followed by the addition of sodium tripolyphosphate reserve liquid, and drug-loaded NPs with the mass ratio of CS and sodium tripolyphosphate at 2:1 were formed until the mixture appeared opalescence. TPP was activated by reacting with EDC and NHS [28] to form semi-stable amine-reactive HNS-ester at constant vibration for 1 h and was further conjugated to the surface of NPs by the formation of stable amide bond. The structure of TPP to CS NPs was investigated by using affinity-1 infrared spectroscopy (Shimadzu, Kyoto, Japan). Nanoparticles were dispersed in distilled water to obtain the homogenous suspension, and the shapes, sizes, and zeta potentials of the particles were determined by a transmission electron microscope (JEM-1200EX; Jeol, Tokyo, Japan) and laser particle analysis (Nano ZS90; Malvern Instruments, Malvern, UK). The encapsulation efficiency of Dox in NPs and drug release characterization in medium with different pH were also evaluated by a previously reported method [29].

### MTT Assay

MTT assay was used to detect antitumor activity of Dox-loaded TPP-NPs in vitro. Briefly, predetermined amount of Dox-loaded NPs were added into distilled PBS buffer at the different volume to obtain a homogenous suspension. Cells were exposed to suspension of NPs at different amounts. Hela cells and A549 cells were seeded in 96-well plates (seeding density was 5 × 10^4^ cells/well) and cultured to logarithmic growth phase. Next, culture medium was removed and re-added serum-free medium respectively containing free Dox and Dox-loaded NPs with different concentrations of Dox into cells for continuous incubation for 24 h. Twenty microliters of MTT (5 mg/mL) was placed into each well to incubate for 4 h followed by the addition of 150 μL of DMSO in each well for dissolving crystal violet precipitation, and the absorbance at 490 nm was detected in a microplate reader.

### Intracellular Distribution and Quantitative Analysis of free Dox and Dox-Loaded TPP-NPs in Cells

In order to investigate the intracellular distribution and uptake intensity of Dox, free Dox and Dox-loaded TPP-NPs were chosen to incubate with A549 cells and Hela cells for observing their internalizing process and quantifying the uptake rate. Briefly, A549 cells and Hela cells at logarithmic growth phase were treated with 0.25% trypsin, and after centrifugation, cell suspension was obtained and seeded in six-well plates at a density of 5 × 10^4^/mL for incubation at 37 °C for 24 h. Then, cells were treated with free Dox and Dox-loaded TPP-NPs containing the same amount of Dox for a certain time. The nucleuses were stained with Hoechst (blue) for 15 min at 37 °C, and the mitochondria were stained by Mitotracker Green FM. Their locations were tracked in cells by confocal laser scanning microscopy (FluoView FV10i; Olympus Corporation, Tokyo, Japan) at given time intervals. Free Dox and Dox-loaded TPP-NPs at the same amount of Dox were treated with both cells, and the intensity of fluorescence from Dox which was excited at 479 nm and emitted at 587 nm was quantified using a microplate reader. The relative uptake ratios of free Dox and Dox-loaded TPP-NPs were determined by calculating the ratio of intracellular fluorescent intensity from internalized free Dox and Dox-loaded TPP-NPs to the initial fluorescent intensity from the total added free Dox and Dox-loaded TPP-NPs at the different time interval.

### Mitochondrial Membrane Potential Change

Cells were digested and seeded into the culture plate to reach a density at cell coverage of 50–70% followed by the separate addition of free Dox and Dox-loaded TPP-NPs. After 24 h, fresh culture medium containing JC-1 at 1 mg/mL was placed into wells and incubated for 30 min at 37 °C under dark conditions. A laser scanning confocal microscope (FluoView FV10i, Olympus, Japan) was used to observe the image change of mitochondrial membrane. JC-1 dye was excited using the 488 nm, and emission light collection was set to 515–545 nm (green) and 570–600 nm (red).

### Intracellular Reactive Oxygen Species (ROS) Measurement

Free Dox and Dox-loaded TPP-NPs were incubated with cells for 12, 24, and 48 h followed by the continuous incubation with 10 μM 2,7-dichlorofluorescein diacetate (DCFH-DA, Sigma, MO, USA) for about 30 min. After washing with PBS, the intracellular DCF fluorescence intensity which was excited at 485 nm and emitted at 530 nm was detected using a microplate reader (Synery-2, Biotek, USA) to investigate the extent of oxidative stress.

### Cell Apoptosis Evaluation by Western Blot

According to the protocol of our previous study [29], free Dox and Dox-loaded TPP-NPs were incubated with cells for 48 h, and then protein supernatant was lysed in RIPA buffer and separated by SDS-PAGE electrophoresis. The protein was transferred from the gel to the PVDF membrane and blocked with 1% BSA at 4 °C overnight. The PVDF membrane was incubated with the primary antibodies (caspase-3, caspase-9, Bax, cytochrome c) at 4 °C overnight, followed by the continuous incubation with secondary antibody and being stained with ECL. The levels of the targeted apoptosis proteins (caspase-3, caspase-9, Bax) and cytochrome c were analyzed by UVP gel analysis system.

## Results

### The Preparation and Characteristics of Various Kinds of NPs

**Fig. 1:**
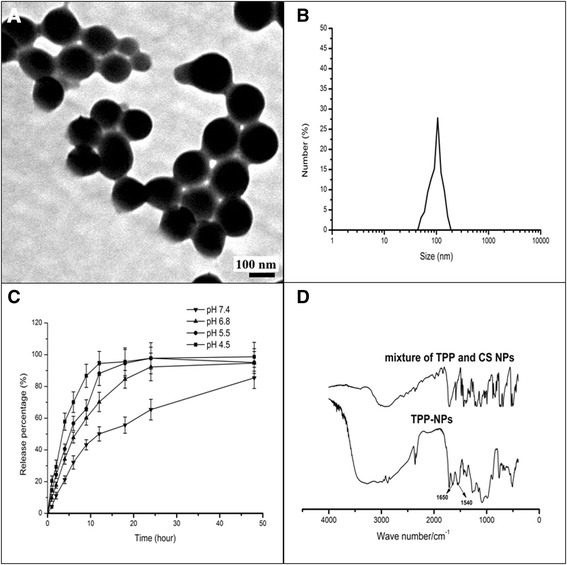
Characterization of Dox-loaded TPP-NPs. **a** TEM image of Dox-loaded TPP-NPs. **b** DLS analysis of the obtained Dox-loaded TPP-NPs. **c** In vitro release profile of Dox-loaded TPP-NPs in phosphate-buffered saline with different pH at 37 °C for 48 h. **d** FT-IR spectra of TPP-NPs and the mixture of TPP and CS NPs

The structural characteristics of the conjugation of TPP to the surface of CS NPs were investigated using IR. It was observed in Fig. 1d that compared with the spectra of the mixture of TPP and CS NPs, the appearance of two intense and sharp peaks at 1650 and 1540 cm^−1^ in the spectra of TPP-NPs indicated that the carboxylic group of TPP molecules and the amino group of CS NPs had reacted to form the new formed amide bond. The particle size and zeta potential of Dox-loaded TPP-NPs were evaluated by Malvern dynamic laser light scattering. The results showed that the average particle size of TPP-NPs was about 100.1 ± 4.3 nm, and the zeta potential was positive and valued at around 15.6 ± 2.3 mV. The morphology of TPP-NPs was spherical in shape and dispersed homogenously with lower polydispersity index at 0.016. In addition, loading efficiency (LE), encapsulation efficiency (EE), and in vitro drug release were also explored. The results indicated that the LE and EE of Dox in TPP-NPs were about 13.2 and 91.3%, respectively. In vitro Dox release experiments (Fig. 1c) indicated that Dox-loaded TPP-NPs showed sustained release of Dox from nanoparticles and varied markedly according to the pH gradient. With the decrease of pH in medium, the releasing rate of Dox was increased and the cumulative release of drug was significantly enhanced, thus enhancing its cytotoxicity in the acidic tumor microenvironment.

### Cellular Viability Study

**Fig. 2:**
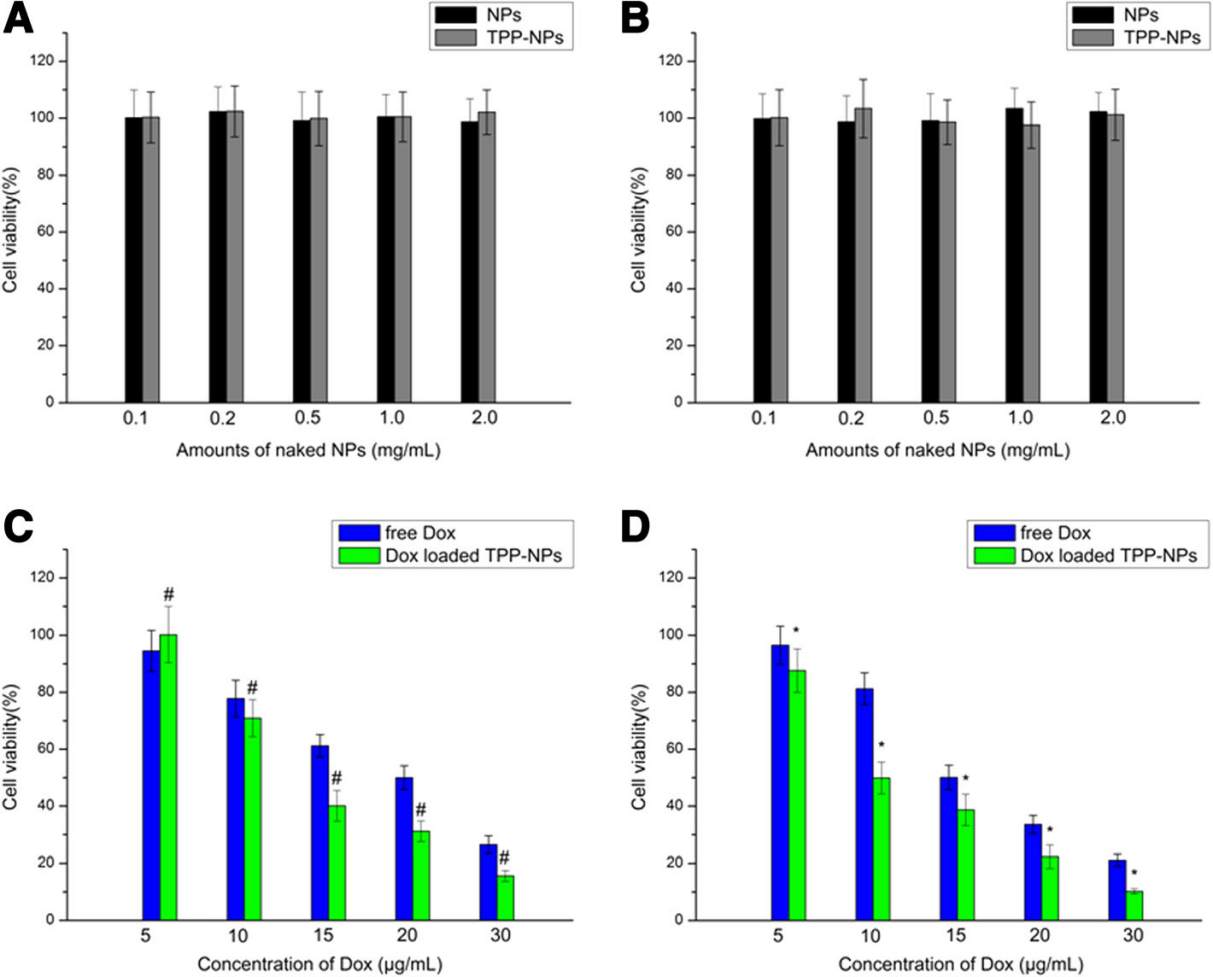
Viability of A549 cells (**a**) and Hela cells (**b**) after incubation with different amounts of naked NPs for 24 h (*n* = 3). Data are presented as means ± SD (*n* = 3). **c** A549 cells viability cultured with Dox-loaded TPP-NPs and free Dox at different concentrations of Dox after 24 h. Data are presented as means ± SD (*n* = 3). #*P* < 0.05 vs the corresponding free Dox. **d** Hela cells viability cultured with Dox-loaded TPP-NPs and free Dox at different concentrations of Dox after 24 h. Data are presented as means ± SD (*n* = 3). *P < 0.05 vs the corresponding free Dox

Cytotoxicities of Dox-loaded TPP-NPs on A549 cells and Hela cells were determined by MTT. As it could be seen from Fig. 2, blank TPP-NPs and CS NPs were not toxic to both cells and the survival rates of TPP-NPs at the amount of 2.0 mg/mL in A549 cells and Hela cells were over 90% within 24 h, suggesting good biological biocompatibility and safety. Compared with free Dox, Dox-loaded TPP-NPs showed significant higher cytotoxicity on both cells in a dose-dependent manner, and the half-maximal inhibitory concentration value of Dox-loaded TPP-NPs treated A549 cells and Hela cells at 24 h was 13.4 and 10.1 μg/mL, with values of 20.3 μg/mL in A549 cells and 15.2 μg/mL in Hela cells for free Dox. These data suggested that Dox encapsulated in TPP-NPs induced the higher cell inhibition effects, and this may be because that with the mediation of TPP for targeting mitochondria, the intracellular distribution of Dox was located at mitochondria instead of accumulating at the nucleus, thus significantly improving the toxicity of Dox in tumor cells.

### In Vitro Cellular Uptake

**Fig. 3:**
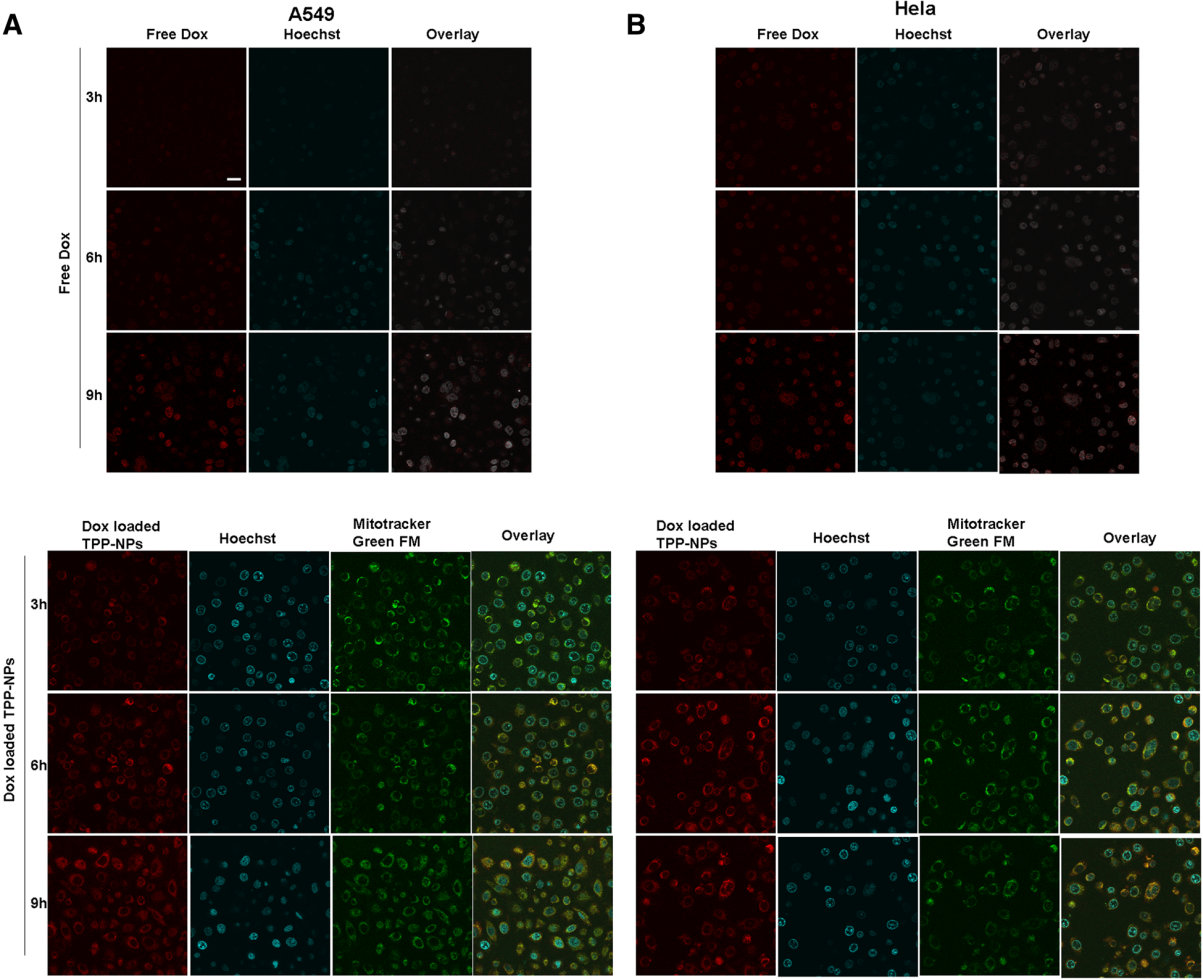
In vitro cellular distribution of NPs after incubating different tumor cells with free Dox and Dox-loaded TPP-NPs. Fluorescent image of A549 (**a**) and Hela cells (**b**), the *scale bar* is 50 μm and applies to all parts

Mitochondrial-targeted ability of Dox-loaded TPP-NPs and time-lapse imaging of intracellular distribution of TPP-NPs in A549 cells and Hela cells were observed by confocal laser scanning microscopy (FluoView FV10i, Olympus, Japan). Generally, Dox was delivered to nucleus and prevented the DNA double helix from being resealed to inhibit the proliferation of tumor cells; therefore, the amount of Dox into the nucleus determined the extent of its antitumor activity. As shown in Fig. 3, when both cells were incubated with Dox, Dox was gradually accumulated in the nuclei of tumor cells and the significant higher red fluorescence emitted by free Dox was focused on the nucleus, indicating that with the passage of time, free Dox was effectively transferred into cell nucleus, thus showing its cytotoxicity. However, when cells were incubated with Dox-loaded TPP-NPs, red spots were homogenously distributed in cytoplasm instead of locating at nucleus, and with the passage of time, the intracellular red fluorescence became stronger and TPP-NPs could be sprinkled throughout the whole cytoplasm. It also demonstrated that in Fig. 3, yellow dots representing co-localization between the inherent red fluorescence of Dox encapsulated in TPP-NPs and the mitochondrial indicator mitotracker® green FM (green) fluorescence were obviously observed, proving that our nanocarrier effectively and specifically delivered its Dox cargo into cell mitochondria and the majority of TPP-NPs were specifically delivered and accumulated in the mitochondria of tumor cells.

**Fig. 4:**
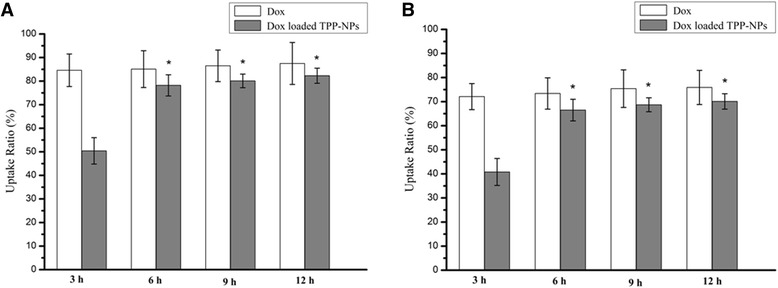
Fluorescence spectrum quantitative analysis of free Dox and Dox-loaded TPP-NPs in A549 (**a**) and Hela cells (**b**). Data are presented as means ± SD (*n* = 3). *P < 0.05 vs Dox-loaded TPP-NPs treated with cells at 3 h

It can be seen from Fig. 4 that when free Dox and Dox-loaded TPP-NPs at the same amount of Dox were treated with both cells, the majority of free Dox had been internalized into cells with the first 3 h and the uptake ratio of free Dox was 84.6% in A549 cells and 72.1% in Hela cells. On the contrary, TPP-NPs showed a slower internalization process and about 50.4 and 40.8% of all NPs were transferred into A549 cells and Hela cells respectively within 3 h. Finally, a majority of NPs accomplished their cellular uptake within 6 h. Generally, free Dox and Dox-loaded TPP-NPs showed a high intracellular uptake ratio in both cells and there were no obvious differences on uptake ability between free Dox and Dox-loaded TPP-NPs.

### Mitochondrial Membrane Potential Analysis

**Fig. 5:**
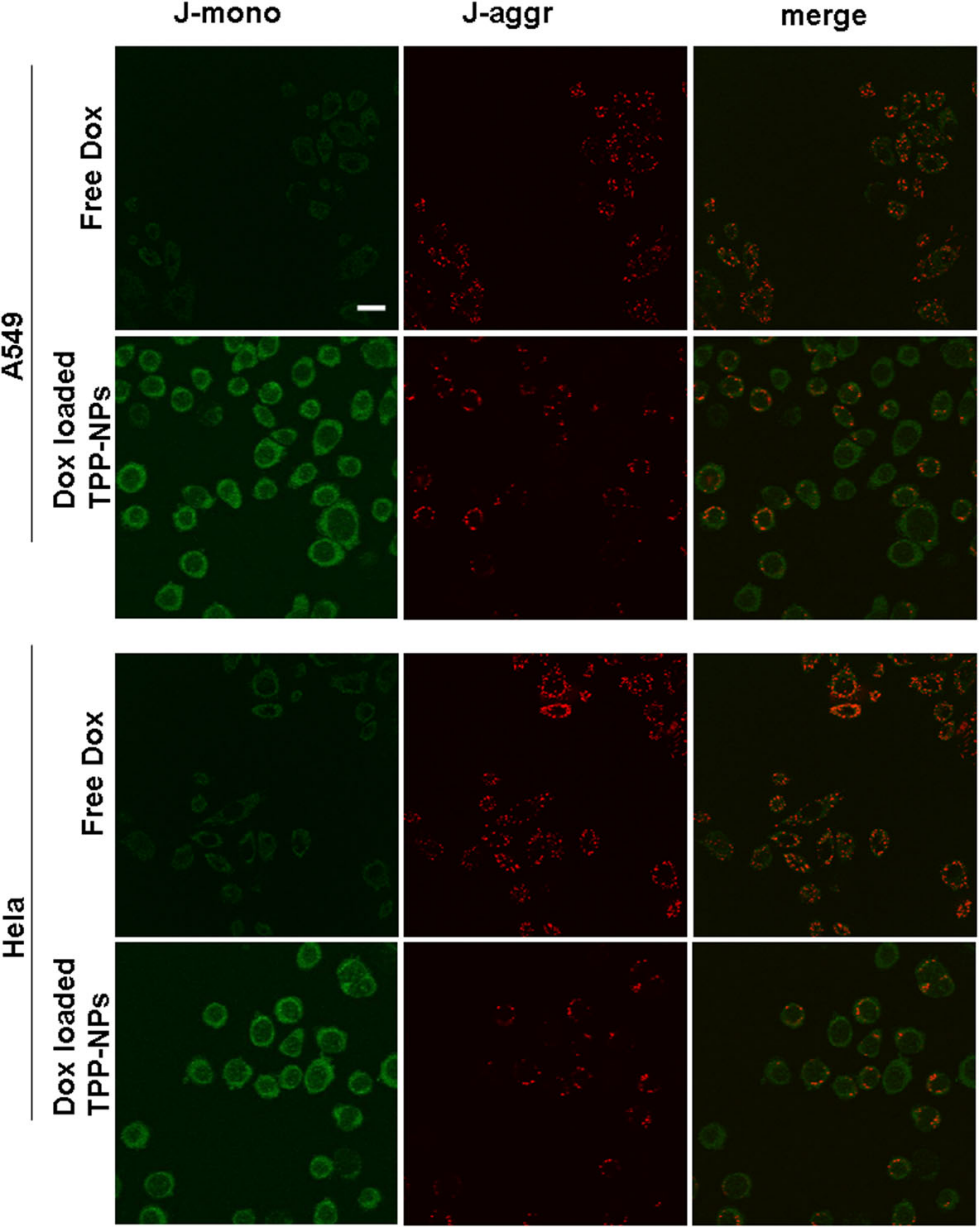
Imaging change of mitochondrial membrane potential after incubating free Dox and Dox-loaded TPP-NPs with A549 cells and Hela cells, the *scale bar* is 50 μm and applies to all parts

It was well known that mitochondria was related with many of the biological activities and especially induced the significant cell apoptosis effects through the loss of mitochondrial membrane potential. Mitochondrial damage led to the depolarization of the mitochondria and caused the decrease of the membrane potential. Changes of mitochondrial membrane potential of the cells after treatment with free Dox and Dox-loaded TPP-NPs were investigated by monitoring the changes of the emitting fluorescence variation of JC-1 dyes. JC-1, as a cationic lipophilic dye, could easily penetrate the cell membrane into the cell and be aggregated in mitochondria with high respiration activity. The aggregation degree of JC-1 was determined by the membrane potential of mitochondria. When cells were in the normal state and the mitochondrial membrane potential was higher (normal), the accumulation of JC-1 in the mitochondria was increased and showed red fluorescence. On the contrary, when the mitochondria were damaged, the membrane potential was decreased and dye molecule aggregation was reduced, thus the dye in the state of a single molecule was dispersed in the cytoplasm and showed green fluorescence. It could be seen from Fig. 5 that when both cells were treated with free Dox, dispersed states of the green fluorescence (JC-1 monomer) and red fluorescent dots (JC-1 aggregation) were both observed, suggesting that the JC-1 dye existed in the single molecular state and the aggregation state. Compared with the weak green fluorescence from free Dox, the change of the intracellular fluorescence of JC-1 in cells treated with Dox-loaded TPP-NPs was observed that red fluorescent intensity was reduced and the intensity of green fluorescence was significantly increased. It also suggested that when cells were treated with Dox-loaded TPP-NPs, the aggregation of JC-1 dye as the indicator of undamaged mitochondrial was reduced and JC-1 monomer as the indicator of damaged mitochondrial was accumulated. The mitochondrial membrane potential was decreased in cells, and the mitochondria in the cells were damaged. The results proved that Dox-loaded TPP-NPs induced the targeted delivery of Dox into mitochondria and significantly enhanced the damage of mitochondria by reducing the mitochondrial membrane potential.

### ROS Determination

**Fig. 6:**
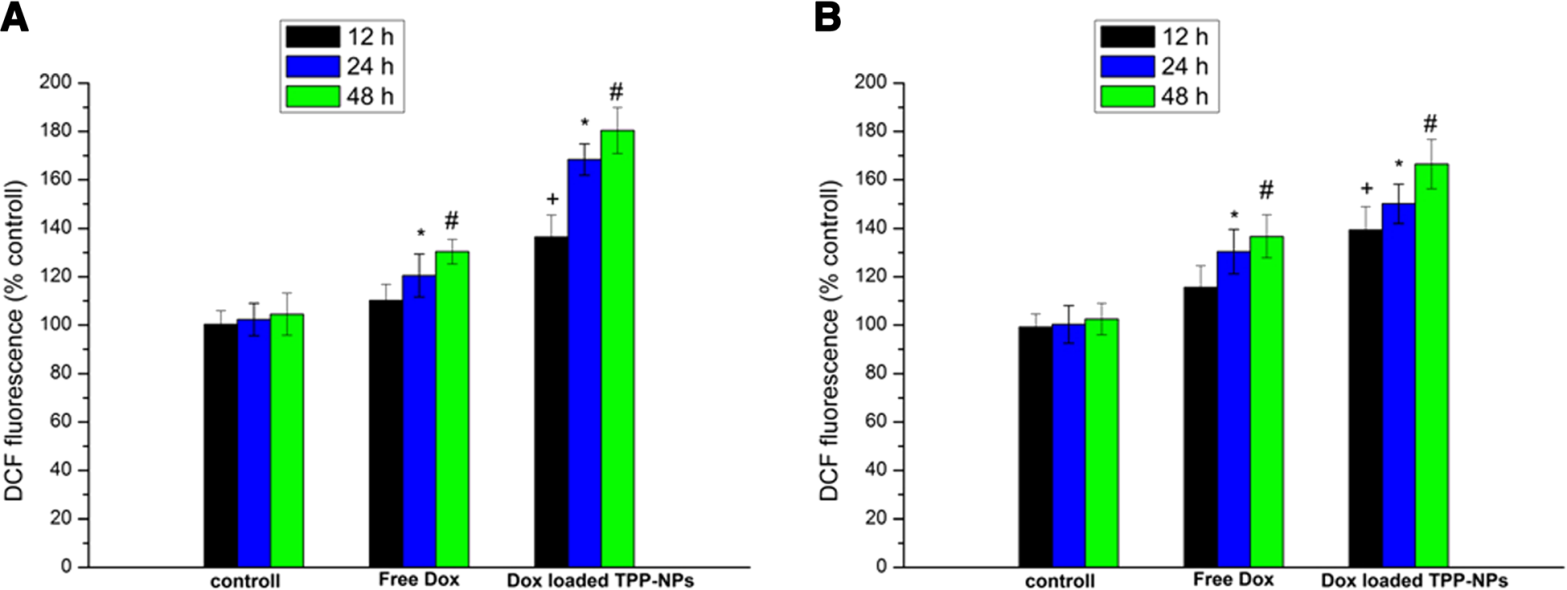
Quantification of ROS generation in cells treated with free Dox and Dox-loaded TPP-NPs at different time. **a** A549 cells. **b** Hela cells. Data are presented as means ± SD (*n* = 3). +*P* < 0.05 vs control group at 12 h, *P < 0.05 vs control group at 24 h, #*P* < 0.05 vs control group at 48 h

The latest study found that ROS played an important role in regulating the process of cell apoptosis, and ROS generation led to mitochondrial swelling and the formation of unspecific channel in mitochondria, thus resulting in the significant decrease of the potential of mitochondrial membrane and further inducing the rapid release of the apoptosis-induced factor and the cytochrome c from mitochondria, finally leading to a series of apoptotic responses. The results (Fig. 6) demonstrated that after treated with free Dox and Dox-loaded TPP-NPs, the generation of ROS in A549 cells and Hela cells was significantly changed. Figure 6a, b demonstrated that ROS levels in A549 cells and Hela cells exposed to free Dox showed a certain enhancement and were increased to 120.5% in 24 h and 130.4% in 48 h in A549 cells in comparison with 130.4% in 24 h and 136.7% in 48 h in Hela cells. Similarly, Dox-loaded TPP-NPs also triggered the augment of ROS levels at 168.4% in 24 h and 180.4% in 48 h in A549 cells, and 150.1% in 24 h and 166.5% in 48 h in Hela cells.

### Western Blot Analysis

**Fig. 7:**
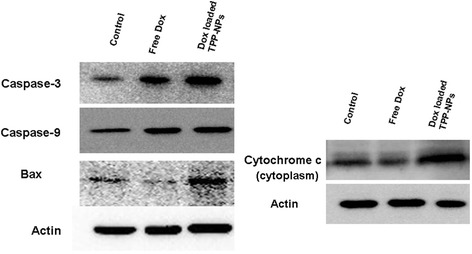
Western blot analyses of the expression levels of caspase-3, caspase-9, Bax, and cytochrome c in Hela cells

It was found that the dysfunction of mitochondria often resulted in cell death, and some mitochondrial apoptosis-related proteins, such as cytochrome c and apoptosis-related proteins, were often regulated. The targeted distribution of Dox in mitochondria induced the rapid release of the apoptosis-inducing factor and the cytochrome c from mitochondria. As shown in Fig. 7, it demonstrated that compared with free Dox, Dox-loaded TPP-NPs with the same concentration of Dox showed the significant enhancement on the expression levels of apoptosis-related proteins such as Bax, caspase-9, and caspase-3, and more cytochrome c were released rapidly from mitochondria into the cytoplasm. It suggested that when Dox was selectively accumulated into mitochondria with the mediation of TPP-NPs, pro-apoptotic protein Bax was activated and the mitochondrial membrane potential was dissipated and further disrupted mitochondrial membrane integrity. Finally, cytochrome c was released rapidly from the mitochondria into cytoplasm and initiated the activation of apoptosis-related proteins such as caspase-9 and caspase-3, eventually inducing significant apoptosis of tumor cells.

## Discussion

Here, we designed a new functional nanoparticle system for enhancing drug accumulation in the mitochondria and leading to the significant apoptosis of cancer cells. The cytotoxicity of Dox caused by the targeted accumulation of Dox mediated by TPP-NPs was enhanced by activating the mitochondria signaling pathway in cancer cells, demonstrating increased activity and the rapid release of cytochrome c, caspase-9, and caspase-3. In order to achieve targeted drug delivery to mitochondria, the cationic lipophilic compound triphenyl phosphine (TPP) had been conjugated to nanoparticles. The results showed that Dox-loaded TPP-NPs were spherical in shape and dispersed homogenously with lower polydispersity index. Dox-loaded TPP-NPs showed sustained release of Dox from nanoparticles and with the decrease of pH in medium; the releasing rate of Dox was increased and the cumulative release of drug was significantly enhanced. At the same time, compared with targeted location of free Dox in nucleus, when cells were incubated with Dox-loaded TPP-NPs, red spots representing Dox were homogenously distributed in mitochondria instead of location at nucleus. The mitochondrial membrane potential was decreased in cells and the mitochondria in the cells were almost completely damaged. After treated with free Dox and Dox-loaded TPP-NPs, the generation of ROS in A549 cells and Hela cells was significantly enhanced, suggesting induction of ROS for triggering the cell apoptosis. Taken together, Dox-loaded TPP-NPs increased the mitochondrial delivery of Dox, therefore increasing cytotoxicity of drug and inducing the significant apoptosis effect.

## Conclusions

We had constructed a mitochondrial-targeted functional nanoparticle delivery system for enhancing antitumor efficiency. The cationic lipophilic compound triphenyl phosphine (TPP) with high mitochondrial binding specificity was introduced, and broad-spectrum anticancer drug Dox was entrapped in the cavity of nanoparticles for achieving the better physiological functions. It showed that Dox-loaded TPP-NPs were internalized into the cytoplasm and further quickly located at the mitochondria to release Dox with the mediation of TPP-NPs. Dox in mitochondria activated the mitochondrial apoptotic pathway, reduced mitochondrial membrane potential, and opened mitochondrial membrane hole, thus releasing cytochrome c and promoting expression of Bax, caspase-3, and caspase-9. Taken together, triphenyl phosphine-conjugated chitosan nanoparticles provided preferential mitochondrial delivery and accumulation of Dox, thus improving drug efficacy in tumor cells.

## References

[1] Bisht S, Maitra A (2009). Dextran-doxorubicin/chitosan nanoparticles for solid tumor therapy. Wiley Interdiscip Rev Nanomed Nanobiotechno.

[2] Szwed M, Wrona D, Kania KD, Koceva-Chyla A, Marczak A (2016). Doxorubicin-transferrin conjugate triggers pro-oxidative disorders in solid tumor cells. Toxicol In Vitro.

[3] Szwed M, Laroche-Clary A, Robert J, Jozwiak Z (2014). Induction of apoptosis by doxorubicin-transferrin conjugate compared to free doxorubicin in the human leukemia cell lines. Chem Biol Interact.

[4] Wu S, Zhao X, Cui Z, Zhao C, Wang Y, Du L, Li Y (2014). Cytotoxicity of graphene oxide and graphene oxide loaded with doxorubicin on human multiple myeloma cells. Int J Nanomedicine.

[5] Foglesong PD, Reckord C, Swink S (1992). Doxorubicin inhibits human DNA topoisomerase I. Cancer Chemother Pharmacol.

[6] Bodley A, Liu LF, Israel M, Seshadri R, Koseki Y, Giuliani FC, Kirschenbaum S, Silber R, Potmesil M (1989). DNA topoisomerase II-mediated interaction of doxorubicin and daunorubicin congeners with DNA. Cancer Res.

[7] Aryal B, Jeong J, Rao VA (2014). Doxorubicin-induced carbonylation and degradation of cardiac myosin binding protein C promote cardiotoxicity. Proc Natl Acad Sci U S A.

[8] Pereira GC, Silva AM, Diogo CV, Carvalho FS, Monteiro P, Oliveira PJ (2011). Drug-induced cardiac mitochondrial toxicity and protection: from doxorubicin to carvedilol. Curr Pharm Des.

[9] Shen RL, Rathe M, Jiang P, Pontoppidan PE, Heegaard PM, Müller K, Sangild PT (2016). Doxorubicin-induced gut toxicity in piglets fed bovine milk and colostrum. J Pediatr Gastroenterol Nutr.

[10] Hayward R, Hydock D, Gibson N, Greufe S, Bredahl E, Parry T (2012). Tissue retention of doxorubicin and its effects on cardiac, smooth, and skeletal muscle function. J Physiol Biochem.

[11] Carvalho C, Santos RX, Cardoso S, Correia S, Oliveira PJ, Santos MS, Moreira PI (2009). Doxorubicin: the good, the bad and the ugly effect. Curr Med Chem.

[12] Xu F, Wang F, Yang T, Sheng Y, Zhong T, Chen Y (2014). Differential drug resistance acquisition to doxorubicin and paclitaxel in breast cancer cells. Cancer Cell Int.

[13] Kopp F, Oak PS, Wagner E, Roidl A (2012). miR-200c sensitizes breast cancer cells to doxorubicin treatment by decreasing TrkB and Bmi1 expression. PLoS One.

[14] Hanušová V, Boušová I, Skálová L (2011). Possibilities to increase the effectiveness of doxorubicin in cancer cells killing. Drug Metab Rev.

[15] Du YZ, Weng Q, Yuan H, Hu FQ (2010). Synthesis and antitumor activity of stearate-g-dextran micelles for intracellular doxorubicin delivery. ACS Nano.

[16] Wang Y, Shi K, Zhang L, Hu G, Wan J, Tang J, Yin S, Duan J, Qin M, Wang N (2016). Significantly enhanced tumor cellular and lysosomal hydroxychloroquine delivery by smart liposomes for optimal autophagy inhibition and improved antitumor efficiency with liposomal doxorubicin. Autophagy.

[17] Szebeni J, Fülöp T, Dézsi L, Metselaar B, Storm G (2016). Liposomal doxorubicin: the good, the bad and the not-so-ugly. J Drug Target.

[18] MacDiarmid JA, Langova V, Bailey D, Pattison ST, Pattison SL, Christensen N, Armstrong LR, Brahmbhatt VN, Smolarczyk K, Harrison MT (2016). Targeted doxorubicin delivery to brain tumors via minicells: proof of principle using dogs with spontaneously occurring tumors as a model. PLoS One.

[19] Wei X, Cohen R, Barenholz Y (2016). Insights into composition/structure/ function relationships of Doxil® gained from “high-sensitivity” differential scanning calorimetry. Eur J Pharm Biopharm.

[20] Hadjidemetriou M, Al-Ahmady Z, Kostarelos K (2016). Time-evolution of in vivo protein corona onto blood-circulating PEGylated liposomal doxorubicin (DOXIL) nanoparticles. Nanoscale.

[21] Mori K, Uchida T, Fukumura M, Tamiya S, Higurashi M, Sakai H, Ishikawa F, Shibanuma M (2016). Linkage of E2F1 transcriptional network and cell proliferation with respiratory chain activity in breast cancer cells. Cancer Sci.

[22] Cheng G, Zielonka J, McAllister D, Hardy M, Ouari O, Joseph J, Dwinell MB, Kalyanaraman B (2015). Antiproliferative effects of mitochondria-targeted cationic antioxidants and analogs: Role of mitochondrial bioenergetics and energy-sensing mechanism. Cancer Lett.

[23] Vyssokikh MY, Antonenko YN, Lyamzaev KG, Rokitskaya TI, Skulachev VP (2015). Methodology for use of mitochondria-targeted cations in the field of oxidative stress-related research. Methods Mol Biol.

[24] Zong WX, Rabinowitz JD, White E (2016). Mitochondria and cancer. Mol Cell.

[25] Kim A (2015). Mitochondria in cancer energy metabolism: culprits or bystanders?. Toxicol Res.

[26] Xiong H, Du S, Ni J, Zhou J, Yao J (2016). Mitochondria and nuclei dual-targeted heterogeneous hydroxyapatite nanoparticles for enhancing therapeutic efficacy of doxorubicin. Biogeosciences.

[27] Zhao L, Yang G, Shi Y, Su C, Chang J (2015). Co-delivery of gefitinib and chloroquine by chitosan nanoparticles for overcoming the drug acquired resistance. J Nanobiotechnology.

[28] Marrache S, Dhar S (2012). Engineering of blended nanoparticle platform for delivery of mitochondria-acting therapeutics. Proc Natl Acad Sci U S A.

[29] Yu X, Yang G, Shi Y, Su C, Liu M, Feng B, Zhao L (2015). Intracellular targeted co-delivery of shMDR1 and gefitinib with chitosan nanoparticles for overcoming multidrug resistance. Int J Nanomedicine.

